# Breast cancer patients’ recall of receiving patient assistance services

**DOI:** 10.1186/2193-1801-1-24

**Published:** 2012-10-03

**Authors:** Jenny J Lin, Kezhen Fei, Rebeca Franco, Nina A Bickell

**Affiliations:** 1 Division of General Internal Medicine, Mount Sinai School of Medicine, One Gustave L. Levy Place, Box 1087, New York, NY 10029 USA; 2 Department of Health Evidence and Policy, Mount Sinai School of Medicine, New York, NY USA

**Keywords:** Recall, Survey research, Breast cancer, Patient assistance

## Abstract

**Background:**

The objective of this study was to assess factors that affect breast cancer patients’ recall of patient assistance services.

**Methods:**

We surveyed newly-diagnosed breast cancer patients and compared recall of receiving patient assistance services at 2 weeks and 6 months in a patient-assistance randomized controlled trial aimed to connect women to such programs. The intervention group received information about assistance programs targeted to their practical, psychosocial, and/or informational needs; the control group received a Department of Health pamphlet about breast cancer and its treatment, including a list of patient assistance services.

**Findings:**

Of 333 women, 210 (63%) reported informational, 183 (55%) psychosocial and 177 (53%) practical needs. At 2 weeks, 96% (202/210) of women with informational needs reported receiving informational material but at 6 months, recall dropped to 69% (140/210). All women whose informational needs were met recalled receiving information, compared to 31% whose needs were unmet (p < 0.0001). Of 109 intervention patients with psychosocial or practical needs, 77% (79) contacted a program specified in their action plan at 2 weeks. However, at 6 months, only 39% (31/79) recalled contacting a program. Women without recall were less likely to report having their needs met (6% vs. 58%; p < 0.001).

**Conclusions:**

Recall of patient assistance services is strongly related to having needs met. Use of patient surveys to evaluate utilization or impact of such programs should be used with caution due to poor patient recall.

**Clinical Trials # NCT00233077:**

http://www.clinicaltrials.gov/ct2/show/NCT00233077?term=Nina+Bickell&rank=2

## Introduction

Since medical chart review is time-consuming, costly and logistically challenging in a decentralized health system, patient self-report via questionnaire surveys are often used to assess utilization of services or treatments (Gordon et al. [1993]; Paskett et al. [1996]). Some studies show patient self-report to be relatively accurate for cancer screening procedures such as pap smears, mammograms and fecal occult blood testing (Gordon et al. [1993]; Zapka et al. [1996]; Mandelson et al. [1999]), cancer treatment (Maunsell et al. [2005]; Clegg et al. [2001]; Oberst et al. [2009]; Liu et al. [2010]; Phillips et al. [2005]; Schootman et al. [2005]), and medication therapy (Boudreau et al. [2004]; Paganini-Hill & [Clark 2007]; Caskie & [Willis 2004]). However, potential problems with patient self-report include inaccurate recall of timing or frequency of screening tests (Bancej et al. [2004]; Caplan et al. [2003a]; Caplan et al. [2003b]; Ferrante et al. [2008]; Howard et al. [2009]) or of details of treatment duration or type (Clegg et al. [2001]; Paganini-Hill & [Clark 2007]). Furthermore, some studies show that cancer survivors may not accurately recall details about their stage of disease, type of tumor, chemotherapy regimens or even having had the disease (Nissen & [Tsai 2011]; Desai et al. [2001]). Accuracy of self-report also appears to decrease with the passage of time (Boudreau et al. [2004]; Craig et al. [2009]).

Efficacy of patient assistance programs, educational or psychosocial interventions is often evaluated, at least in part, by questionnaires (Fiscella et al. [2011]; Freund et al. [2008]; David et al. [2011]; Allicock et al. [2010]). These surveys often ask participants to rate satisfaction with, utilization of or effectiveness of program services. As participants may have inaccurate recollections of their use of such programs and interventions, it is important to assess factors that affect patient recall. Accurate recall becomes particularly important if future funding decisions are based in part on patients’ recall of service utilization. Moreover, as many studies evaluating health care utilization are based on survey data, it is important to assess the reliability of self-reported data collected by survey questionnaire. This study was part of a randomized controlled trial to evaluate whether connecting women with newly-diagnosed breast cancer with patient-assistance services could improve use of adjuvant therapies. In this paper, we evaluate newly-diagnosed breast cancer patients’ recall of informational materials received and patient assistance programs contacted.

## Methods

### Setting and participants

Women with early-stage breast cancer who were eligible for post-surgical adjuvant therapy were recruited from 8 New York City hospitals (4 municipal, 4 tertiary referral centers). Women were recruited within 2–4 weeks after definitive surgical treatment and were block-randomized to intervention or usual care. Women who did not speak English or Spanish or could not provide informed consent were excluded. The study was approved by the IRBs of all 8 participating hospitals.

### Study design & intervention

We surveyed participants at baseline and 6 months about their experiences with care, health status, social support, self-efficacy, knowledge, attitudes and beliefs about breast cancer and its treatment, patient assistance programs contacted and help received from programs. Scales for health status (SF-12), social support (Strogatz et al. [1997]), and medical mistrust (LaVeist et al. [2000]) were scored to 100 with higher values indicating higher health status, social support, and physician trust, respectively. For all patients, we conducted a baseline survey measuring breast cancer experiences, knowledge, attitudes and beliefs, and a needs assessment. For intervention patients, we identified 3 high-quality patient assistance programs that could address each patient’s identified need and created an individualized action plan with each patient to enable contact with the patient assistance programs. A hard copy of the action plan and related print materials were mailed. Patients in the control group were sent a pamphlet about breast cancer and its treatment created by the New York State Department of Health that also included resources’ contact information. Two weeks later, we called all patients to confirm receipt of mailed materials; intervention patients were also asked whether they had connected with the programs specified in their action plan. If they did not connect with a patient assistance program and still had psychosocial or practical needs, we assigned an outreach worker to help them connect with a program. At 6 months, we surveyed patients to determine the type of help they had received from the patient assistance program and how helpful it was. A patient was classified as having gotten her needs met if she reported receiving information, counseling or practical help for her identified need.

### Analysis

Summary statistics were used to describe baseline demographics, and chi-square tests for categorical variables, T-tests or ANOVA for continuous variables to assess group differences. Logistic regression models were fit to assess risk factors of the primary outcome, 6-month recall of patient assistance services. All analyses were performed using SAS version 9.2, with the type 1 error rate fixed at 0.05 (2 tailed).

## Results

Of the 333 women who completed the 6 month follow-up survey, 210 (63%) reported informational needs. At 2 weeks, 96% (202/210) reported receiving the breast cancer information pamphlet but at 6 months, only 69% (140/210) recalled receiving informational material (Table 1). Of the 140 who recalled getting informational material, 80% reported they had their informational needs met. All 112 women who had their informational needs met recalled getting informational material (100%; p < 0.001), compared to 31% of those who did not have their informational needs met. Of the 109 women in the intervention group with psychosocial or practical needs who were told of relevant assistance programs, 79 (77%) contacted the patient assistance program specified in their action plan at 2 weeks. However, at 6 months, only 31/79 (39%) recalled contacting a program. Women who did not recall contacting a program at 6 months were less likely to have had their psychosocial or practical needs met (6% vs. 58%; p < 0.001). Age, income, race, education, language spoken, social support, adjuvant treatment, trust in physicians and physical or emotional health status did not affect either recall of informational material or programs contacted.

**Table 1:** **Factors associated with recall among women with informational needs & women with practical or psychosocial needs**

	Recalled Informational help (N = 140)	Did not recall Informational Help (N = 62)	P	Recalled Practical or Psychosocial Help (N = 31)	Did not recall Practical or Psychosocial help (N = 48)	P
Age (yrs) mean ± SD	57 ± 11.9	58 ± 10.0	0.62	56 ± 10.6	56 ± 12.1	0.78
Race			0.35			0.49
Black	30 (22%)	19 (32%)		7 (24%)	14 (30%)	
White	45 (34%)	18 (31%)		9 (31%)	9 (19%)	
Hispanic	59 (44%)	22 (37%)		13 (45%)	24 (51%)	
High school graduate	102 (73%)	45 (73%)	0.97	20 (65%)	34 (71%)	0.56
Insurance			0.36			0.78
Medicare	21 (15%)	13 (21%)		4 (13%)	9 (19%)	
Medicaid/Non-Insured	51 (36%)	17 (27%)		14 (45%)	21 (44%)	
Commercial	68 (49%)	32 (52%)		13 (42%)	18 (38%)	
Income < $15,000/year	46 (33%)	19 (31%)	0.76	11 (35%)	17 (35%)	0.99
English speaking	81 (58%)	39 (63%)	0.50	18 (58%)	22 (46%)	0.29
Needs met	112 (80%)	0	<0.001*	18 (58%)	3 (6%)	<0.001*
Radiation treatment received	93 (67%)	45 (73%)	0.42	21 (68%)	34 (71%)	0.77
Chemotherapy received	88 (63%)	34 (56%)	0.31	20 (65%)	30 (63%)	0.86
Hormonal therapy received	99 (71%)	48 (79%)	0.27	24 (77%)	34 (71%)	0.52
Trust MD, mean ± SD^+^	94 ± 8.4	92 ± 8.2	0.07	93 ± 9.1	92 ± 9.7	0.68
Instrumental Social Support, mean ± SD^+^	67 ± 27.3	70 ± 24.4	0.48	71 ± 29.5	67 ± 25.2	0.50
Emotional Social Support, mean ± SD^+^	79 ± 19.3	79 ± 18.8	0.83	77 ± 21.2	78 ± 19.6	0.91
SF-12, Physical Health, mean ± SD^+^	44 ± 11.7	46 ± 10.3	0.20	45 ± 9.0	44 ±11.4	0.53
SF-12, Mental Health, mean ± SD^+^	49 ± 11.4	46 ± 10.1	0.08	48 ± 13.4	48 ± 12.4	0.93

^+^Scores scaled to 100.

In multivariable analysis adjusting for age, education and receipt of chemotherapy, women who had their psychosocial or practical needs met were almost 4 times more likely (95% confidence interval: 2.35 - 6.55) to recall having contacted a patient assistance program compared to women who did not have their needs met (Table 2).

**Table 2:** **Multivariable model predicting recall of practical and psychosocial help**

Covariates	RR (95% Confidence Interval)	P
Age	1.00 (0.98 - 1.03)	0.65
High school education	0.88 (0.56 - 1.38)	0.57
Chemotherapy received	1.23 (0.75 - 2.02)	0.41
Practical/psychosocial needs met	3.93 (2.36 - 6.55)	<0.001

Model c-statistics = 0.778; p < 0.001.

## Discussion

Surveys are widely used to assess patients’ experiences, beliefs and knowledge about medical care and treatment. While surveys are an efficient method to collect data, they may not always be accurate or reliable. We found that breast cancer patients’ recall of information about patient assistance programs was not associated with an individual patient’s psychological or physical state but rather on the direct relevance and impact of the data and programs on their lives. Nearly all of the women who received informational materials about breast cancer and treatment recalled getting this information at 2 weeks, but this proportion dropped substantially by 6 months. Strikingly, their recall of having received informational material was significantly associated with whether they reported that their informational needs had been met.

Even more striking was the poor recall of practical and psychosocial services in our study. Of the women with underlying psychosocial or practical needs, more than three-quarters report contacting patient assistance programs initially, but by 6 months, only a third of the women who had contacted a program recalled doing so. Similar to recall of informational material, recall of contacting a program was also strongly related to whether women felt their needs had been met.

Patients who have better recall report more satisfaction with physician communication (Gabrijel et al. [2008]) and greater trust of their providers (Posma et al. [2009]). Additionally, patients’ accurate recall of medical information can directly affect adherence with prescribed treatment regimens ([Kessels 2003]; Watson & Mc[Kinstry 2009]; Pickney & [Arnason 2005]) and can indirectly provide signals about patients’ experiences and the quality of those experiences. Moreover, recall of services utilized may impact patients’ reported satisfaction and thus affect quality improvement efforts and possibly reimbursements tied to quality ratings (van Campen et al. [1995]; [Kravitz 1998]; Turnbull & [Luther 1996]; Epstein et al. [2004]; [Takach 2011]; [Bickell 2999]) From a research perspective, asking patients about services utilized offers a less expensive assessment approach than conducting chart reviews, particularly for care that may be fragmented and not accessible electronically. Studies find patient report to be reliable for cancer treatments received (Oberst et al. [2009]; Phillips et al. [2005]; Schootman et al. [2005]; Penfold et al. [2011]), yet the reliability of patient report to assess utilization is variable (Lubeck & [Hubert 2005]) with greater concerns among vulnerable patients (Sohler et al. [2009]). Our study calls into question the accuracy of self-reported utilization of patient assistance services among breast cancer patients. Any inferences made about underutilization of services reported in survey data using patient self-report should be treated with caution.

Numerous conditions can affect the accuracy of patient recall. Individuals who are older, sicker, less educated and have more negative emotional or psychological states have worse recall of information presented (Jansen et al. [2008]; Ong et al. [2000]; Liang et al. [2002]; Butow et al. [1998]). In addition to the many personal attributes that can challenge an individual’s recollection, recall also depends on the effective communication of accurate information so it can be conveyed and subsequently recalled. These challenges are great in everyday circumstances but can be even more acute for patients with cancer, a diagnosis fraught with physical and psychological threats. Unlike prior studies, however, we found that recall of information was not affected by individual factors such as age, educational level, emotional status, or type of adjuvant therapy received. In our study, only whether patients felt their needs had been met by the services provided affected recall accuracy.

There are a number of limitations to this study. Because this was a study of women receiving breast cancer care in an urban setting, our findings may not generalize to women receiving care in other settings. We did not assess the quality of the contact that women had with patient assistance programs, although the patient assistance programs were high-quality programs specifically chosen based on previous work evaluating quality of patient assistance programs in New York City (Cohen et al. [2009]). Moreover, we did not assess contact with patient assistance programs among the usual care group at the 2 week phone call and are unable to assess their true level of utilization. Thus, it is quite possible that our measure of recall is an underestimate. Still it is striking that of the 79 women who did contact a patient assistance program, only a third of them recalled doing so at 6 months, even after adjusting for age, education and receipt of chemotherapy, which might affect memory (Koppelmans et al. [2012]).

Accuracy of breast cancer patients’ survey responses about patient assistance program utilization is strongly related to their perception of the usefulness of the services received. Our findings should make researchers and others take pause regarding use of patient report as a reliable source to assess program utilization since it appears that recall was closely related to patients’ perceptions of whether the program helped them.
